# Intermittent Vibration Induces Sleep via an Allatostatin A‐GABA Signaling Pathway and Provides Broad Benefits in Alzheimer's Disease Models

**DOI:** 10.1002/advs.202411768

**Published:** 2024-12-10

**Authors:** Yang Mou, Yan Zhang, Yuxian Zheng, Guang He, Zhi‐Xiang Xu, Xiao Xiao, Yong Ping

**Affiliations:** ^1^ Bio‐X Institutes Key Laboratory for the Genetics of Developmental and Neuropsychiatric Disorders (Ministry of Education) Shanghai Jiao Tong University Shanghai 200240 China; ^2^ State Key Laboratory of Medical Neurobiology MOE Frontiers Center for Brain Science, and Institutes of Brain Science Fudan University Shanghai 200032 China; ^3^ Key Laboratory of Computational Neuroscience and Brain‐Inspired Intelligence Ministry of Education Behavioural and Cognitive Neuroscience Center Institute of Science and Technology for Brain‐Inspired Intelligence MOE Frontiers Center for Brain Science Fudan University Shanghai 200433 China

**Keywords:** allatostatin A, alzheimer's disease, GABA, sleep, vibration

## Abstract

While animals across species typically experience suppressed consciousness and an increased arousal threshold during sleep, the responsiveness to specific sensory inputs persists. Previous studies have demonstrated that rhythmic and continuous vibration can enhance sleep in both animals and humans. However, the neural circuits underlying vibration‐induced sleep (VIS) and its potential therapeutic benefits on neuropathological processes in disease models remain unclear. Here, it is shown that intermittent vibration, such as cycles of 30 s on followed by 30 s off, is more effective in inducing sleep compared to continuous vibration. A clear evidence is further provided that allatostatin A (AstA)‐GABA signaling mediates short‐term intermittent vibration‐induced sleep (iVIS) by inhibiting octopaminergic arousal neurons through activating GABA_A_ receptors. The existence of iVIS in mice is corroborated, implicating the GABAergic system in this process. Finally, intermittent vibration not only enhances sleep but also reduces amyloid‐β (Aβ) deposition and reverses memory defects in Alzheimer's disease models. In conclusion, the study defines a central neural circuit involved in mediating short‐term iVIS and the potential implications of vibration in treating sleep‐related brain disorders.

## Graphical abstract

This study introduces a novel vibration modality that effectively induces sleep in both *Drosophila* melanogaster and mice. Evidence from the research indicates that the AstA‐GABA signaling pathway plays a crucial role in mediating sleep induced by intermittent vibration, highlighting the potential of vibration therapy for addressing sleep‐related brain disorders.

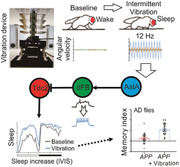

## 1 Introduction

Sleep, a phenomenon conserved across diverse animal species, poses a challenge to our understanding due to its complex regulation and multifaceted functions.^[^ ^1^, ^2^ ^]^ Random mechanical shaking and slamming forced wakefulness was conventionally used to deprive sleep in experimental animals,^[^ ^2^, ^3^ ^]^ and this strong mechanical stimulation may cause physiological and behavioral effects that are independent of sleep as well.^[^ ^4^ ^]^ Several reports have demonstrated that gentle and rhythmic mechanical vibration or rocking can enhance sleep in a variety of organisms, including flies, mice, and humans.^[^ ^5^, ^6^, ^7^, ^8^, ^9^, ^10^, ^11^, ^12^, ^13^ ^]^ These findings align with the notion that certain sensory processing mechanisms for external stimuli remain active during sleep, potentially enabling more prompt reactions,^[^ ^14^, ^15^, ^16^ ^]^ or even contributing to enhanced sleep under specific circumstances.^[^ ^17^ ^]^ Despite these observations, the neural circuits governing vibration‐induced sleep (VIS) remain elusive.

The quantity and quality of sleep play pivotal roles in maintaining overall health.^[^ ^18^, ^19^ ^]^ Notably, sleep has been shown to contribute to the clearance of debris in the brain, cognition, and mental health.^[^ ^20^, ^21^, ^22^, ^23^, ^24^, ^25^ ^]^ However, nearly half of the adults over 60 years old are suffering from sleep disturbance, which may be a candidate risk factor for neurological diseases, including Alzheimer's disease (AD).^[^ ^26^, ^27^, ^28^ ^]^ Although several treatment options are available for sleep disturbance in the elderly population,^[^ ^29^ ^]^ achieving effective treatment remains a considerable challenge. It is of particular interest to explore whether non‐invasive interventions, such as gentle vibration, can positively impact sleep and memory, especially under specific neuropathological conditions.

In this study, we measured the potential impacts of mechanic vibration in different conditions on sleep in flies and mice and delineated relevant central neural circuits mediating intermittent vibration‐induced sleep (iVIS). We further explored whether iVIS is functional and its potential physiological significance.

## 2 Results

### 2.1 Intermittent Vibration Enhances Sleep in *Drosophila*

**Figure 1:**
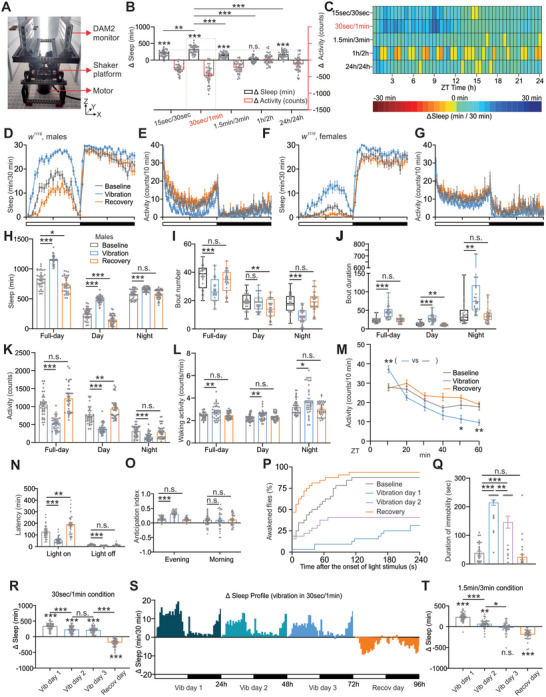
iVIS in flies. A) Vibration device. DAM modules were mounted on a platform, which was connected to a vibrating motor. B) Sleep time changes for one day in different vibration conditions, including, 15s/30s, 30s/1 min, 1.5min/3min, 1h/2h, and 24h/24 h (n = 42–46 flies). Sleep time and activity count changes were calculated as vibration day minus baseline day. C) Sleep change heatmaps for the data shown in (B). Note that a long gap in 1h/2h mode causes sleep reduction during vibration‐off periods. D–G) Sleep and activity profiles of *w^1118^* flies in 12 h light/dark (LD) including baseline, vibration, and recovery days. Males (D, E, n = 39) and females (F, G, n = 36) are included. White and black bars indicate 12 h LD periods, respectively. H–M) Quantification of full‐day, daytime, and nighttime sleep amounts (H), bout number (I), bout duration (J), activity counts (K), waking activity (L), and activity for 60 min after vibration on (M) in males as shown in (D, E). Quantification data for females are shown in Figure S1 (Supporting Information). Quantification for baseline, vibration, and recovery data were all shown, as indicated. N,O) Quantification of sleep latency (N) and anticipation index (O) in males as shown in (D). Sleep latency and anticipation index for both lights on and off were shown. P) Percentage of WT flies that start moving (defined as awakened) in response to bright light within 4 min under four conditions, including baseline, vibration on day 1, vibration on day 2, and the recovery day. Note that there is spontaneous awakening in the absence of light stimuli (at 0 s). Q) Quantification of the duration of immobility in response to 1‐min bright light stimulus within 4 min as shown in (P). n = 32 flies. R,S) Quantification of sleep time change (R) and sleep time change profile (S) for three‐day intermittent vibration in 30s/1 min condition under 12 h LD cycles. Sleep time change for recovery (Recov) day was also shown. Vibration (Vib) induced an increase in sleep sustained for up to three days and there was a negative rebound during the recovery day. n = 41 flies. T) Quantification of sleep time change for three‐day continuous vibration at 1.5min/3min mode. Sleep increment was gradually reduced and there is no significant difference on day 3. n = 38 flies. Error bars indicate SEM. n.s., not significant, ^*^ *p* < 0.05, ^**^ *p* < 0.01, ^***^ *p* < 0.001. Statistical significance in (B) was assessed by two‐way ANOVA with Sidak's multiple comparison test. For multiple comparisons in (H, K‐O, Q, R, and T), statistical significance was assessed by one‐way ANOVAs followed by post hoc Tukey test. Box plots were used for bout number and duration in (I, J), and statistical significance was assessed by the Mann–Whitney U test. The line inside the box indicates the median; the upper and lower box limits the 75 and 25% quantiles; vertical lines above and below the box represent the 90 and 10% quantiles; points show the 95 and 5% outliers.

To investigate the regulatory effects of gentle vibration on sleep, we mounted the *Drosophila* Activity Monitoring (DAM) module on a custom‐made platform, which was connected to a gently vibrating motor (**Figure** **1**A). Three types of motors, each with different vibrating rates, were tested, revealing a stable 3D motion primarily along the Y‐axis on the DAM2 module for each motor. Notably, we found that 12 Hz vibration produced robust sleep increase under normal 12h/12h light/dark (LD) cycles in *w^1118^* males (Figures S1A,B, Supporting Information).

Five vibration conditions were applied, including cycles of 15 s of vibration followed by 15 s of rest (15 s/30 s), cycles of 30 s of vibration followed by 30 s of rest (15 s/1 min), cycles of 1.5 min of vibration followed by 1.5 min of rest (1.5min/3min), cycles of 1 h of vibration followed by 1 h of rest (1h/2 h) and continuous vibration throughout the entire day (24h/24 h). We observed increased total sleep time in 15 s/30 s, 30 s/1min, 1.5min/3min, and 24h/24 h vibration setups at 12 Hz rate (Figures 1B,C). Intriguingly, the most pronounced enhancement in sleep occurred in the 30 s/1 min vibration condition compared to the others. Consequently, we selected the 12 Hz vibrating rate and the 30 s/1min condition for further analysis.

As expected, sleep exhibited a significant increase during intermittent vibration for one day, with a notable extension in bout duration, particularly evident in daytime sleep for wild‐type (WT, *w^1118^*) males (Figures 1D,E,H,I,J). iVIS was similarly observed in females, with relevant data presented in Figures 1F,G, and S1C–I (Supporting Information). Although general activity counts were decreased, waking activity was not reduced (Figures 1E,K,L), indicating iVIS was not caused by defects of locomotor ability. Notably, we observed a negative sleep rebound during the daytime on the following recovering day (Figure 1H), suggesting iVIS is potentially functional by accumulating sleep credit. Reports have indicated that abdominal tremulations from a courting male and looming stimuli may cause immobility and a freezing (or fleeing) response.^[^ ^30^, ^31^ ^]^ However, we observed that male flies initially exhibited a surge in activity within the first 10 min after the onset of vibration, followed by a gradual decline (Figure 1M), suggesting that the male flies exhibited fleeing to the vibration onset and took a few minutes to habituate. Similar results were also obtained from WT females (Figure S1J), Supporting Information. Vibration also led to shortened sleep onset and increased evening anticipation index (Figures 1N,O).

Flies in the sleep monitoring box without motor‐on exhibited normal sleep and activity (Figures S1K,L, Supporting Information), indicating that mild noise produced by nearby motors has minimal effects on sleep patterns. To assess whether iVIS is deeper than baseline sleep, we measured the arousal threshold by calculating the percentage of flies awakened in response to bright light stimuli. Vibration significantly increased the duration of immobility of flies in response to a 1‐min bright light stimulus (Figures 1P,Q), indicating light responsiveness is reduced during vibration.

We further showed that iVIS can last for several days in WT flies (Figures 1R,S), indicating its potential implication as sleep innervation. We noted that iVIS was completely absent on the third day of vibration in 1.5min/3min condition (Figure 1T), highlighting the critical importance of appropriate vibration conditions for inducing robust and long‐lasting sleep.

### 2.2 AstA and GABA Signaling is Involved in iVIS

**Figure 2:**
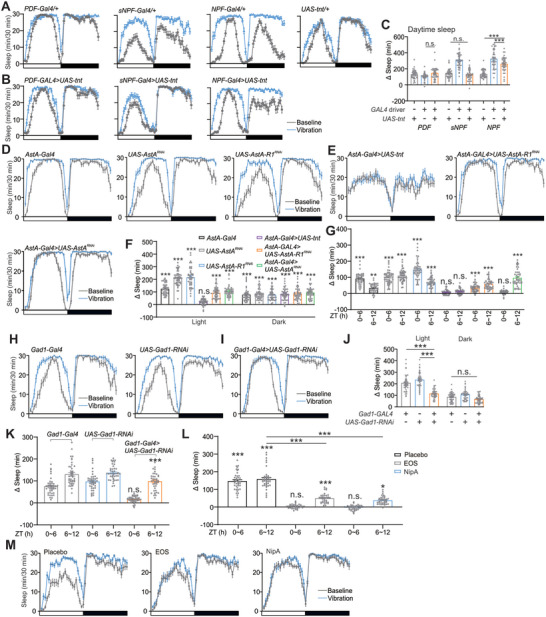
GABA signaling is involved in short‐term iVIS. A,B) Conventional sleep profiles of controls (A) and experimental flies (B) of indicated genotypes in 12 h LD conditions. Baseline and vibration were shown in gray and blue, respectively. C) Quantification of daytime sleep changes as shown in (A, B). n = 40–42 flies for each case. D,E) Conventional sleep profiles of controls (D) and experimental flies (E) of indicated genotypes in 12 h LD conditions. F,G) Quantification of daytime and nighttime sleep changes (F) as shown in (D, E). Sleep time changes during ZT 0–6 and 6–12 was also shown (G). n = 41–50 flies for each case. H,I) Sleep profiles of controls (H) and experimental flies (I) of indicated genotypes in 12 h LD conditions. J,K) Quantification of daytime and nighttime sleep changes (J) as shown in (H, I). Sleep time changes during ZT 0–6 and 6–12 was also shown (K). n = 42–43 flies for each case. L) Quantification of sleep time changes during ZT 0–6 and 6–12 in WT flies fed with EOS or NipA under 12 h LD. n = 36–42 flies for each case. M) Sleep profiles of placebo (control) and drugs (EOS or NipA) treatment in WT flies were shown in 12 h LD conditions. Error bars indicate SEM. n.s., not significant, ^*^ *p* < 0.05, ^**^ *p* < 0.01, ^***^ *p* < 0.001. Statistical significance in (C, F, G, J, K, M) was assessed by One‐way ANOVAs followed by the post hoc Tukey test.

To explore the neural circuits by which vibration enhances sleep, we genetically silenced parts of sleep‐regulating neurons by using the *Gal4/UAS* binary expression system. Numerous neuropeptides, co‐released with neurotransmitters in flies, were found to regulate sleep, rhythmic activity, and metabolic homeostat,^[^ ^2^, ^32^, ^33^ ^]^ so we intended to examine whether specific well‐defined peptides play a role in iVIS. We first used *PDF‐, sNPF‐* or *NPF‐Gal4* to drive tetanus toxin (*UAS‐tnt*) to block synaptic output from these peptide‐expressing neurons. Unfortunately, these lines showed normal daytime iVIS compared to control lines (**Figures** **2**A–C and S2A, Supporting Information), indicating that theses peptide‐expressing neurons are not involved in daytime iVIS. Although nighttime iVIS was blocked in the *sNPF‐Gal4>UAS‐tnt* line, we propose this could be due to a ceiling effect (Figures 2B and S2A, Supporting Information). Since flies typically sleep more during nighttime, we focused on daytime sleep in the following experiments to avoid the potential ceiling effect.

Furthermore, visual and circadian clock mutants, including *GMR‐hid*, *NinaC*,^[^ ^3^ ^]^ *PDF^01^* and *per^KG00546^*, showed normal iVIS (Figures S2B–F, Supporting Information). Blocking synaptic activity in different subsets of mushroom body neurons also failed to block iVIS (Figure S2G, Supporting Information). Fortunately, we found that inhibiting synaptic output of another neuropeptide allatostatin A (AstA)‐expressing neurons (*AstA‐Gal4>UAS‐tnt*) blocked daytime iVIS (F_(6301)_ = 139.5, *AstA‐Gal4>UAS‐tnt*, vibration versus baseline, n.s. p = 0.4288), while nighttime iVIS persisted (Figures 2D–G). Given that *AstA‐Gal4* labels groups of neurons in the central brain (Figure S2H, Supporting Information),^[^ ^34^ ^]^ blocking the activity of AstA neurons would impact multiple neural circuits. We next asked whether AstA signaling mediated daytime iVIS. Interestingly, inhibiting AstA expression in AstA neurons (*AstA‐Gal4>UAS‐AstA^RNAi^*) specifically blocked iVIS at Zeitgeber time (ZT) 0–6, while downregulating AstA receptor 1 (AstA‐R1) expression in AstA neurons (*AstA‐Gal4>UAS‐AstA‐R1^RNAi^*)) left iVIS unaffected (Figures 2D–G), indicating AstA signaling in the downstream of AstA neurons, but not in AstA neurons, is essential for short‐term iVIS (ZT 0–6). The efficacy of *AstA* RNAi was validated through qPCR, which demonstrated a reduction in *AstA* mRNA levels by over 80% (Figure S2I, Supporting Information), supporting AstA signaling is required for short‐term iVIS.

Since a previous report suggested that GABA signaling is involved in 6‐h VIS in *Drosophila*,^[^ ^5^ ^]^ we reduced GABA signaling by knocking down *glutamic acid decarboxylase 1* (*Gad1*), an enzyme essential for GABA synthesis, using *GAD1‐Gal4* and *UAS‐Gad1^RNAi^*. As expected, the knockdown of *GAD1* specifically blocked iVIS at ZT 0–6 (Figures 2H–K), aligning with the results from the AstA knockdown experiments. These results indicate that the biological basis of iVIS can be dissected into short‐term (ZT 0–6) and long‐term iVIS (ZT 6∼), with these two phases potentially uncoupled from each other.

To test whether the increase in overall GABA levels can also block short‐term iVIS, we took a pharmacological approach by feeding WT flies with drugs to block GABA transaminase (ethanolamine‐O‐sulphate, EOS) or GABA transporter (nipecotic acid, NipA), thereby augmenting overall GABA levels in the fly brains. We found that short‐term iVIS was specifically blocked when treated with EOS or NipA (Figures 2L,M). We also observed that both EOS and NipA treatments dramatically increased daytime sleep, reaching levels comparable to those observed under conditions of intermittent vibration (Figures 2L and S2J, Supporting Information). This increase in sleep duration could potentially inhibit iVIS, possibly due to a ceiling effect where the sleep‐inducing capacity of the drugs approaches the maximum physiological limit (Figures 2L,M).

### 2.3 GABA Released by dFB Neurons mediates iVIS During ZT 0–6

**Figure 3:**
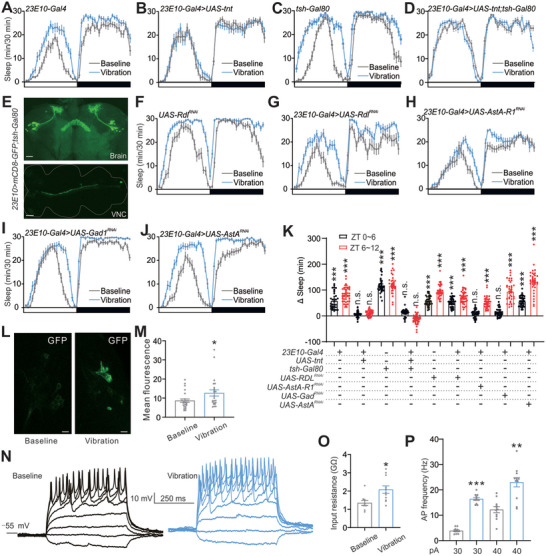
GABA in dFB neurons is involved in iVIS. A–D) Sleep profiles of controls (A, C) and experimental flies (B, D) of indicated genotypes in 12 h LD conditions. E) Representative confocal stack of the brains and VNC from the indicated genotype stained with GFP antibody. Scale bars, 20 µm. F–J) Sleep profiles of controls (F) and experimental flies (G–J) of indicated genotypes in 12 h LD conditions. K) Quantification of sleep time changes during ZT 0–6 and 6–12 as shown in (E‐I). n = 36–44 flies for each case. L) Representative confocal image of baseline and vibration of *23E10‐Gal4>UAS‐CaLexA* stained with GFP antibody. Flies were dissected immediately after vibration (from ZT 0–3) at ZT3. Scale bar, 5 µm. M) Quantification of GFP fluorescence intensity in 23E10 neurons at baseline and vibration conditions as shown in (L). n = 24 for baseline and 23 for vibration. N) Representative voltage responses to 500 ms current steps (from ‐10 to 60 pA, 10 pA increment per step) of *23E10‐Gal4>UAS‐mCD8‐GFP* at baseline and vibration conditions. O,P) Quantification of input resistance (O) and firing rates (P) as recorded in (N). n = 9–13 cells for each case. Error bars indicate SEM. n.s., not significant, ^*^ *p* < 0.05, ^**^ *p* < 0.01, ^***^ *p* < 0.001. Statistical significance in (M, O) was assessed by Student's t‐test. Statistical significance in (K, P) was assessed by one‐way ANOVAs followed by the post hoc Tukey test.

One group of AstA neurons, identified as circadian pacemaker neurons, also contributes to sleep‐promoting effects of the dorsal fan‐shaped body (dFB neurons) probably by acting on AstA receptors.^[^ ^35^ ^]^ Additionally, dFB neurons were found to regulate sleep by releasing GABA acting on GABA receptors in octopaminergic arousal neurons.^[^ ^35^ ^]^ Thus, we tested the potential contribution of AstA and GABA signaling in dFB neurons to iVIS. Accordingly, we used *23E10‐Gal4* to drive *UAS‐tnt* to block synaptic output from dFB neurons. Strikingly, we found that full‐day iVIS was almost completely blocked (**Figure** **3**A,B,K). Although activation of dFB neurons was suggested to promote sleep or regulate sleep homeostasis in flies,^[^ ^36^, ^37^, ^38^ ^]^ recent work has raised questions regarding the precise role of these neurons, and possible involvement of ventral nerve cord (VNC) neurons.^[^ ^39^, ^40^ ^]^ We used *tsh‐Gal80*, which blocked Gal4 activity under the control of the *tsh* promotor (mainly in VNC).^[^ ^40^ ^]^ To our surprise, adding *tsh‐Gal80* was unable to restore iVIS in the *23E10‐Gal4*>*UAS‐tnt* line (Figures 3C,D,K), indicating activities of dFB neurons are required for iVIS. We also confirmed dFB neurons labeled by *23E10‐Gal4>UAS‐mCD8‐GFP;tsh‐Gal80*, and a few labeled neurons in VNC (Figure 3E).

Furthermore, we showed that either downregulation of AstA‐R1 or reducing GABA content by downregulating *Gad1* in dFB neurons specifically blocked iVIS during ZT 0–6, while downregulation of GABA_A_ receptor gene, *Resistant to dieldrin* (*Rdl*), by expression of *Rdl^RNAi^* with *23E10‐Gal4* exerted no effect on iVIS (Figures 3F–K), demonstrating that activation of AstA‐R1 in dFB neurons and subsequent release of GABA are essential for short‐term iVIS.

To further evaluate the role of dFB in iVIS, we applied CaLexA (calcium‐dependent nuclear import of LexA) to examine if vibration during ZT 0–3 could induce sustained activity. 23E10 neurons show a higher GFP signal immediately after vibration for 3 h compared to baseline controls (Figures 3L,M), confirming the dFB neurons are more active during vibration. Finally, we took patch‐clamp recordings in GFP‐labeled 23E10 neurons from isolated brains to test their excitability after vibration. We used current injection to elicit membrane voltage responses in dFB neurons and found that more action potentials (APs) were elicited immediately after vibration for 3 h from ZT 0 to 3 compared to baseline controls (Figures 3N–P), in agreement with the CaLexA data. Moreover, dFB neurons during vibration appeared to be more active with lower input resistance (Figure 3O).

### 2.4 GABA Receptors in Octopaminergic Neurons are Involved

Octopaminergic neurons, especially for subgroups of neurons that reside in the protocerebrum named anterior superior medial (ASM) neurons, exert wake‐promoting effects.^[^ ^41^ ^]^ dFB neurons release GABA as sleep‐promoting neurotransmitters acting on downstream targets including octopaminergic neurons.^[^ ^35^ ^]^ We next asked whether there are functional GABA_A_ receptors in octopaminergic neurons, and if so, whether they mediate iVIS during ZT 0–6.

**Figure 4:**
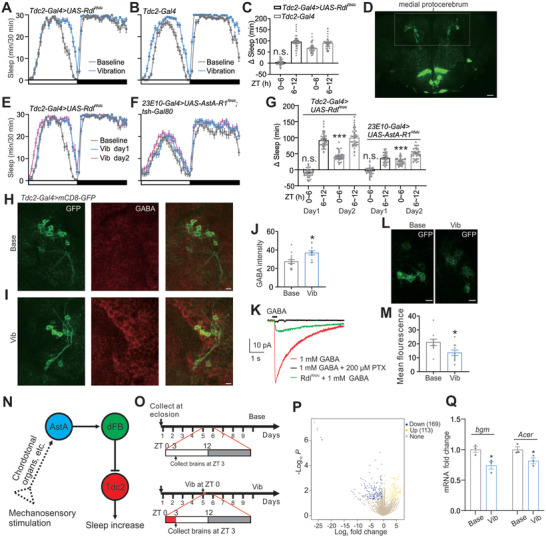
GABA_A_ receptors in Tdc2 neurons mediate short‐term iVIS. A,B) Sleep profiles of controls (B) and experimental flies (A) of indicated genotypes in 12 h LD conditions. C) Quantification of sleep time changes during ZT 0–6 and 6–12 in fly lines as shown in (A, B). n = 39–43 flies for each case. D) Expression pattern of the *Tdc2‐GAL4* line as visualized with an mCD8‐GFP reporter. Labeled neurons in dashed lines are a subgroup residing medial protocerebrum (or named ASM neurons). Scale bar, 20 µm. E,F) Sleep profiles of indicated genotypes in baseline, vibration (vib) day 1, and vib day 2. G) Quantification of sleep time changes during ZT 0–6 and 6–12 in fly lines as shown in (E, F). n = 33–45 flies for each case. H,I) Brains of the indicated genotype dissected at ZT3 including baseline (Base, H) and vibration (Vib, I) for 3 h from ZT 0–3 is immunostained with GABA (red). Scale bars, 5 µm. J) Bar graphs represent relative GABA intensity as shown in (H, I). n = 12–14 brains for each case. K) Representative recording traces mediated by GABA_A_ receptors obtained from GFP‐labeled neurons in medial protocerebrum. L) Representative confocal image of ASM neurons including baseline and vibration of *Tdc2‐Gal4>UAS‐CaLexA* stained with GFP antibody. Flies were dissected immediately after vibration (from ZT 0–3) at ZT3. Scale bar, 5 µm. M) Quantification of GFP fluorescence intensity in ASM neurons at baseline and vibration conditions as shown in (L). n = 10 brains for baseline and vibration. N) Model. O) Schematic representation of RNA‐sequencing design. P) Volcano plot representing the distribution of DEGs under vibration compared to the baseline condition. DEGs with statistical significance were selected with the criteria of |log2‐fold change| ≥ 0.58 (≥ 1.5‐fold changes) and FDR < 0.05. Q) Shown are RT‐qPCR analyses for mRNA levels of *bgm* and *Acer* in baseline (control) and vibration conditions. n = 3 independent qPCR for each case. Error bars indicate SEM. n.s., not significant, ^*^ *p* < 0.05, ^**^ *p* < 0.01, ^***^ *p* < 0.001. Statistical significance in (J, M) was assessed by Student's t‐test. Statistical significance in (C, G, Q) was assessed by one‐way ANOVAs followed by post hoc Tukey test.

As expected, downregulation of Rdl (GABA_A_ receptor subunit) in octopaminergic neurons (driven by *Tdc2‐Gal4*) specifically blocked iVIS at ZT 0–6 (**Figures** **4**A–D), suggesting GABA released from dFB neurons acting on Rdl in Tdc2 neurons mediates short‐term iVIS.

To examine whether short‐term iVIS was specific to ZT 0–6, we subjected transgenic lines lacking short‐term iVIS (*Tdc2‐Gal4>UAS‐Rdl^RNAi^* or *23E10‐Gal4>UAS‐AstA‐R1^RNAi^;tsh‐Gal80*) to vibration for two days (Figures 4E,F). Our data showed that vibration increased sleep during ZT 0–6 on the second day of vibration (Figure 4G), indicating that the mutants blocked short‐term iVIS independent of clock time.

We next examined the effects of vibration on GABA levels around ASM neuronal soma by immunostaining. GABA levels at ASM were significantly increased after vibration for 3 h (ZT 0–3) (Figures 4H–J), and the increase in GABA seems not a widespread phenomenon across the entire brain (Figure S3A, Supporting Information). Additionally, GABA levels in dFB neurons were not significantly altered by vibration (Figures S3B,C, Supporting Information). To confirm the GABA_A_ receptor‐mediated currents in ASM neurons labeled by GFP (Figure 4D, from posterior sections), we performed voltage‐clamp recordings. Robust GABA currents were observed and were blocked by a GABA_A_ receptor blocker (picrotoxin, PTX) (Figure 4K). The currents were also dramatically reduced by downregulating Rdl expression through RNAi (Figure 4K), demonstrating there are functional GABA_A_ receptors in ASM neurons. We also applied CaLexA to examine if vibration during ZT 0–3 could induce sustained inactivity. As expected, ASM neurons show a lower GFP signal immediately after vibration for 3 h compared to baseline control (Figures 4L,M).

### 2.5 Vibration Downregulates Two Metabolic Genes

We next wondered if GABA signaling‐mediated short‐term iVIS would influence gene expression across the whole brain and if it belongs to “active” or “quiet” (deep) sleep as described in recent reports.^[^ ^22^, ^42^ ^]^ We induced vibration in *w^1118^* flies for 3 h from ZT 0 to ZT 3 and collected samples for RNA‐seq (Figure 4M). 282 genes in total were included as differentially expressed genes (DEGs), with more genes (169) downregulated and fewer genes (113) upregulated (Figure 4N and Table S1, Supporting Information). We further processed these DEGs by Gene Ontology (GO) analysis. Several GO biological processes (BP) are altered, notably proteolysis (Table S1, Supporting Information). This aligns with recent findings suggesting a potential link between proteostasis and sleep, both of which are characteristics of aging and neurodegenerative diseases.^[^ ^43^, ^44^ ^]^ We also noted that the primary metabolic process is slightly but significantly altered, with two downregulated metabolic genes including bgm (bubblegum, FBgn0027348) and Acer (angiotensin‐converting enzyme‐related, FBgn0016122), which were further confirmed by qPCR results (Figure 4O and Table S1, Supporting Information), indicating that short‐term iVIS may also alter metabolic rate.

### 2.6 Mice Exhibited iVIS, which was Reduced by Flumazenil

**Figure 5:**
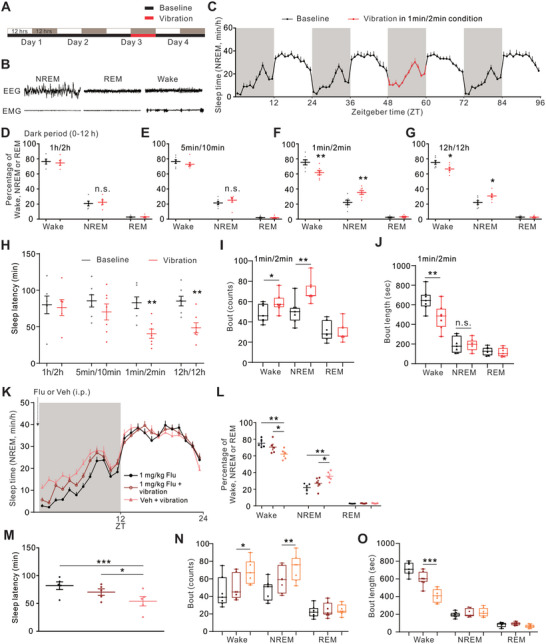
iVIS in mice. A,B) Schematic representation of vibration design for mice polygraphic recordings (A) and typical EEG/EMG recording traces during wakefulness, NREM sleep, and REM sleep (B). C) Time course for NREM sleep (Mean, min/h) in baseline or vibration conditions for WT mice. We only vibrate the animals for 12 h (during dark periods) as shown in red lines. See also Table S2 (Supporting Information). D–G) Percentage of time in NREM sleep, REM sleep, or wake state during dark period in baseline (the day before vibration, shown in gray) and subsequent in four conditions of vibration (red), including 1h/2h (cycles of 1 h vibration followed by 1 h rest) (D), 5min/10min (cycles of 5 min vibration followed by 5 min rest) (E), 1min/2min (cycles of 1 min vibration followed by 1 min rest) (F) and 12h/12h (continuous vibration for 12 h) (G). n = 7 mice for each case. H) Quantification of sleep latency (dark period) under baseline and vibration conditions. I,J) Bout counts (I) and bout duration (J) during wake, NREM, and REM sleep during a dark period in baseline or vibration (in 1min/2min condition). K) Time course for NREM sleep in baseline or vibration (during the dark period) conditions in the groups of WT mice with vehicle (Veh) or Flu injected intraperitoneally (i.p.) 15 min prior to the vibration onset as indicated. L) Percentage of time in NREM sleep, REM sleep, or wake state during dark period in baseline or vibration conditions as shown in (K). n = 5 mice for each case. M) Quantification of sleep latency (dark period) under baseline or vibration conditions as shown in (K). N,O) Bout counts (N) and bout duration (O) of wake, NREM sleep, and REM sleep in the dark period under baseline or vibration conditions as shown in (K). Error bars indicate SEM. n.s., not significant, ^*^ *p* < 0.05, ^**^ *p* < 0.01, ^***^ *p* < 0.001. Statistical significance in (H) was assessed by two‐way ANOVA with Sidak's multiple comparison test. For multiple comparisons in (D–G, L, M), statistical significance was assessed by one‐way ANOVAs followed by post hoc Tukey test. Box plots were used for bout number and duration in (I, J, N, O), and statistical significance was assessed by Mann–Whitney U test.

To test whether iVIS is conserved across animal species, we examined the possible effects of mechanic vibration on sleep in mice. We placed C57BL/6N WT mice on a rhythmic and laterally moving platform (Figure S4A, Supporting Information, see also Experimental Section). Following surgery, mice were given 1–2 weeks to recover before simultaneous recording of electromyography (EMG) and electroencephalogram (EEG) was conducted over the subsequent four days to monitor different sleep stages (**Figures** **5**A,B). According to a previous report,^[^ ^7^ ^]^ we subjected the mice to 1 Hz vibration for 12 h to induce sleep.

Accordingly, vibration in four different conditions was applied during the dark period on the third day (Figures 5C–G, Table S2, Supporting Information). Intermittent vibrations in 1h/2h (cycles of 1 h vibration followed by 1 h rest) and 5min/10min (cycles of 5 min vibration followed by 5 min rest) conditions showed no significant effects on wakefulness, NREM and REM sleep as compared to the baseline condition (Figures 5D,E and Table S2, Supporting Information). Interestingly, 1min/2min vibration significantly increased NREM sleep, accompanied by reduced wake time, while REM sleep was unaltered (Figures 5C,F,G). Continuous vibration (12h/12 h) also increased NREM sleep but was quantitatively lower compared to 1min/2min vibration (Figure S4B, Supporting Information). Vibration also shortened NREM sleep latency in 1min/2min and 12h/12h conditions, but unaltered for 1h/2h and 5min/10min vibration (Figure 5H). Thus, we chose 1min/2min vibration for further analysis. We further found that the NREM bout number was increased, while the NREM bout duration remained unchanged during vibration (Figures 5I,J). These data suggest that intermittent vibration induces sleep in WT mice similar to iVIS in *Drosophila*, although the effects of vibration on sleep were more robust in flies with a larger amount of sleep increase and enhanced bout duration.

To determine whether signaling pathways mediating *Drosophila* iVIS are conserved, we evaluated the possible involvement of the GABAergic system in mice. We pretreated mice with flumazenil to specifically block GABA_A_‐benzodiazepine receptors, as reported previously.^[^ ^45^ ^]^ iVIS was significantly reduced by injection of 1 mg kg^−1^ flumazenil, while vibration still induced a comparable increase in NREM sleep and shortened sleep latency in the presence of 0.1 mg kg^−1^ flumazenil (Figures 5K,L and S4C–E, Table S2, Supporting Information), indicating flumazenil blocks iVIS in a dose‐dependent manner. 1 mg kg^−1^ flumazenil injection alone showed no significant effect on NREM and REM sleep in mice (Figure S4F, Supporting Information). As expected, shortened sleep latency and increased bout number by vibration were also diminished by 1 mg kg^−1^ flumazenil (Figures 5M–O).

### 2.7 Enhanced Sleep by Vibration in AD Models

**Figure 6:**
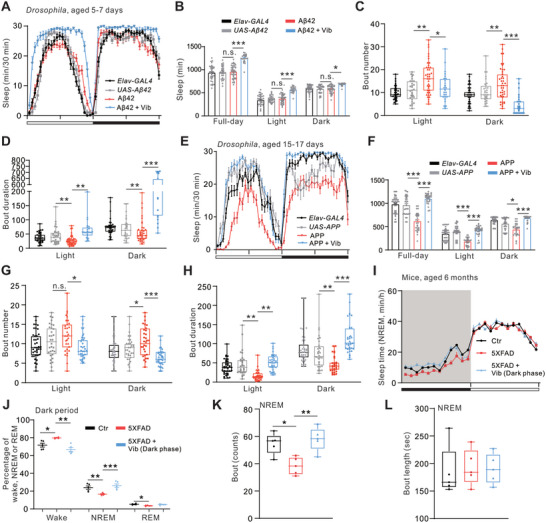
Vibration enhances sleep in AD models. A) Sleep profiles of indicated *Drosophila* genotypes in 12 h LD conditions. Vibration was applied for a full‐day in Aβ42 flies (*Elav‐Gal4>UAS‐Aβ42*). B) Quantification of full‐day, daytime, and nighttime sleep as shown in (A). n = 36–44 flies for each case. C,D) Quantification of bout number (C) and bout duration (D) for daytime and nighttime in lines as shown in (A). E) Sleep profiles of indicated *Drosophila* genotypes in 12 h LD conditions. Vibration was applied for a full‐day in APP flies (*Elav‐Gal4>UAS‐APP*). F) Quantification of full‐day, daytime, and nighttime sleep as shown in (E). n = 32–43 flies for each case. G,H) Quantification of bout number (G) and bout duration (H) for daytime and nighttime in lines as shown in (E). I) Time course for NREM sleep (Mean, min/h) during baseline and vibration for control (Ctr) and 5XFAD transgenic mice as indicated. Vibration (Vib) was applied for 12 h during the dark phase. J) Percentage of time in NREM sleep, REM sleep, or wake state during dark period in lines as shown in (I). n = 5 mice for each case. K,L) Bout counts (K) and bout duration (L) for NREM sleep during dark periods in lines as shown in (I). Error bars indicate SEM. n.s., not significant, ^*^ *p* < 0.05, ^**^ *p* < 0.01, ^***^ *p* < 0.001. Statistical significance in (B–J) was assessed by two‐way ANOVA with Sidak's multiple comparison test. Box plots were used for bout number and duration in (C, D, G, H, K, L), and statistical significance was assessed by the Mann–Whitney U test.

Next, we asked whether intermittent vibration could enhance sleep in AD animals, including *Elav‐Gal4>UAS‐Aβ42* (Aβ42), *Elav‐Gal4>UAS‐APP* (APP) flies and 5XFAD mice, all of which exhibited sleep disorders as reported previously.^[^ ^46^, ^47^, ^48^, ^49^, ^50^ ^]^ Aβ42 flies showed sleep fragmentation (increased in sleep bout number) without changes in sleep time. 24‐h vibration increased sleep time and bout duration, and decreased bout number (**Figures** **6**A–D), demonstrating that vibration reverses sleep fragmentation in the AD flies. Vibration also enhanced sleep in APP flies by increasing sleep time and bout duration (Figures 6E–H), similar to that observed in Aβ42 flies.

For 3‐month‐old 5XFAD mice, no difference was found in the time spent in wakefulness, NREM, and REM sleep compared to the control mice (Figures S5A–D, Supporting Information). 6‐month‐old 5XFAD showed an increase in wake duration, accompanied by a decrease in NREM and REM sleep during the dark phase, while no difference was observed during the light phase (Figures 6I,J). Interestingly, vibration during the dark phase enhanced sleep in 5XFAD mice at both ages, largely due to an increase in NREM sleep (Figures 6I,J and S5A,B, Supporting Information). Vibration preferentially increased bout number, but not bout duration, in 5XFAD mice (Figures 6K,L), similar to that observed in WT mice.

### 2.8 Aβ Deposition and Memory Defects were Reversed by Vibration

**Figure 7:**
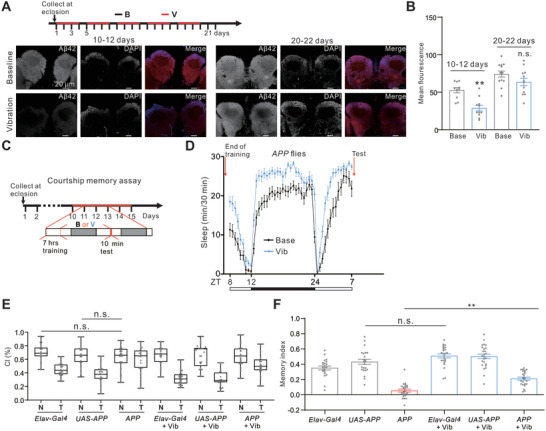
Vibration ameliorates neuropathological processes in AD flies. A) Schematic representation of vibration design (top) and representative immunostaining for Aβ42 in Aβ42 flies at indicated ages. Vibration was applied for three out of four days. B) Quantification of Aβ42 fluorescence intensity in the mushroom body of baseline (Base) and vibration (Vib) groups as shown in (A). n = 10–13 flies for each case. C) Schematic representation of vibration and long‐term courtship memory design. Experimental APP flies were vibrated (V) after 7‐h training for one day. B, Baseline. D) Sleep profiles of APP fly after training under baseline or vibration conditions. We finished training at ZT 7 and measured the activity of the flies from ZT 8. n = 38–43 flies for each case. E) Courtship index (CI) of the lines as indicated. n = 21–25 groups for each case. N, naïve; T, trained. F) Memory index (MI) of the lines as indicated. n = 21–25 groups for each case. Error bars indicate SEM. n.s., not significant, ^*^ *p* < 0.05, ^**^ *p* < 0.01, ^***^ *p* < 0.001. Statistical significance in (B, F) was assessed by one‐way ANOVAs followed by the post hoc Tukey test. Box plots were used for CI in (E), and statistical significance was assessed by the Mann–Whitney U test.

Since there is a potential bidirectional relationship between sleep and AD neuropathology,^[^ ^26^, ^27^, ^28^, ^51^ ^]^ we set out to reveal whether vibration alleviates AD‐related neuropathological processes. 30 s/1min vibration caused a long‐lasting increase in sleep in Aβ42 flies for up to 9 days (Figure S6A, Supporting Information), so we implemented a protocol involving cycles of 3‐day (d) vibration followed by 1‐d rest in flies after eclosion (**Figure** **7**A). Vibration greatly reduced Aβ42 levels in the mushroom body (MB) area at the age of 10–12 d (Figures 7A,B), indicating sustained iVIS enhances Aβ42 clearance. Unexpectedly, no difference in Aβ fluorescence in flies aged 20–22 d. We observed numerous degenerative “holes” as reported previously,^[^ ^52^ ^]^ (Figure 7A) and this could reduce general Aβ fluorescence, making it challenging and inaccurate for Aβ quantification in aged flies.

Reports have suggested that acute Gaboxadol (THIP)‐induced “deep” sleep could reverse courtship memory decline in a *Drosophila* AD model,^[^ ^53^ ^]^ and also in aged flies.^[^ ^54^ ^]^ The courtship conditioning assay was conducted to evaluate long‐term courtship memory under both baseline and vibration conditions. The memory index (MI) was calculated by determining the difference between the courtship index (CI) of trained (T) male flies and that of naïve (N) male flies, followed by dividing this difference by the CI of the naïve flies, as detailed in the Experimental Section. We found that MI was greatly reduced in APP flies aged ≈12 d, while CI was not affected in APP flies compared to control lines (Figures 7C–F). We introduced vibration after training and sleep was indeed greatly enhanced during the following day (Figures 7D and S6B–D, Supporting Information). Using *23E10‐Gal4>UAS‐CD8‐GFP* line, we also confirmed increased firing rate in dFB neurons at ZT 9–10 (vibration for ≈2 h), but not for 10 h vibration (Figures S6E,F, Supporting Information), suggesting AstA‐GABA signaling contributes to short‐term iVIS in the memory test. Furthermore, we assessed whether vibration could reverse memory defects in AD models using a long‐term courtship memory assay. MI was greatly increased by vibration in APP flies, but not for baseline controls (Figure 7F). Similar results were also observed in Aβ42 flies (Figure S6G, Supporting Information).

## 3 Discussion

To unravel the intricacies of sleep regulation and function, reliable methods for inducing and controlling sleep in animals are crucial. Our study highlights the efficacy of intermittent vibration in inducing quantitatively more sleep compared to continuous vibration in both *Drosophila* and mice, presenting a promising avenue for future sleep induction research. We defined an AstA‐GABA signaling pathway mediating VIS at ZT 0–6. Notably, the mutants with blocked VIS at ZT 0–6 tended to exhibit full‐day VIS (including ZT 0–6) on the second day of vibration (Figure 4). Our data also showed that hyperactivity in dFB neurons is a short‐term effect when vibration starts at ZT 7.5 (Figures 7 and S6, Supporting Information). In sum, we propose that blocking VIS at ZT0≈6 in the mutants has a short‐term effect, independent of clock time. Interestingly, dopamine signaling in the PAM cluster was suggested to regulate behavioral responsiveness to a series of strong mechanical vibrations (or arousability) in flies,^[^ ^55^ ^]^ indicating gentle and strong mechanical vibrations exert opposite effects on sleep via distinct signaling pathways.

Recent studies have suggested that the activation of chordotonal organs mediates mechanosensory stimulus‐induced sleep, and GABA signaling‐mediated habituation, a form of simple learning, plays a role in VIS in flies,^[^ ^5^, ^6^ ^]^ but the linking chordotonal organs and GABA signaling to sleep circuitry remains elusive. In this work, we delineate a central neural circuit – composed of activating GABA release by AstA‐R1 signaling in dFB neurons and subsequent inhibition of wake‐promoting octopamine neurons by activation of GABA_A_ receptors – that controls short‐term iVIS (ZT 0–6) (Figure 4N). If habituation plays a crucial role in iVIS, our data suggested it could be separated into short‐term and long‐term habituation (STH & LTH). Indeed, the two forms were indicated in previous studies, and mechanisms underlying STH and LTH were suggested to differ from each other.^[^ ^56^, ^57^ ^]^ Our data support GABA signaling system is involved in mediating STH, but not for LTH.

Our pharmacological findings showed that overall enhancement of brain GABA levels by EOS and NipA significantly attenuated iVIS, with a complete blockade of short‐term iVIS. At the cellular level, the drugs‐induced increase in GABA are hypothesized to persistently activate a significant portion of GABA receptors. This constant activation could potentially lead to receptor desensitization or inactivation, thereby reducing the availability of GABA receptors that can respond when additional GABA is released in the dFB neurons in response to vibration. Additionally, both drugs increased daytime sleep to levels comparable to those observed under vibration conditions, potentially creating a ceiling effect that diminishes iVIS.

Although AstA neurons were found to inhibit feeding behavior and promote sleep in flies,^[^ ^34^, ^35^, ^58^, ^59^, ^60^ ^]^ the specific regulation of sleep by AstA signaling remains unclear.^[^ ^35^, ^58^, ^61^ ^]^ Our data showed that general downregulation of AstA expression in AstA neurons (driven by *AstA‐Gal4*, related data shown in Figure 2) showed no obvious effects on sleep time in adult males. Since part of VNC neurons are labeled by *23E10‐Gal4*, the specific roles of AstA receptors on sleep in dFB subgroups need further study.^[^ ^61^ ^]^ Additionally, dFB neurons are activated by several neurotransmitters including glutamate,^[^ ^35^ ^]^ so whether glutamate signaling is changed by AstA and its potential roles in iVIS warrants further investigation. Multisensory inputs, including mechanosensory stimulus, were found to regulate feeding behavior in *Drosophila*,^[^ ^62^, ^63^ ^]^ as well as sleep reported in this study. These reports indicate that there is a potential interrelationship between mechanosensory stimulus and sleep centers, and the neuropeptide AstA could act as a molecular signaling hub. Additional research is needed to uncover the upstream neural circuits of AstA neurons and elucidate how AstA neurons are regulated during vibration.

Interestingly, the vestibular system was identified to be required for gentle rocking‐induced sleep in mammals,^[^ ^7^ ^]^ yet the downstream sleep‐regulating center remains elusive. In this work, we confirmed that GABA signaling plays a conserved role in mediating iVIS from flies to mammals, but specific brain regions involved need further study. Interestingly, vestibular inputs might influence sleep‐wake state through several brain structures, including the orexigenic lateral hypothalamus.^[^ ^64^ ^]^ Activities of orexin neurons were found to be inhibited by GABA, which could be released from the ventrolateral preoptic nucleus (VLPO) and other neurons.^[^ ^65^ ^]^ A recent report suggested that there was a potential reciprocal inhibitory relationship between the VLPO and hypothalamic arousal orexin neurons via GABA signaling.^[^ ^66^ ^]^ More importantly, a subpopulation of VLPO neurons contains the neuropeptide galanin (homologous to AstA in *Drosophila*), which was found to be involved in mediating iVIS in *Drosophila* in this work. Whether galanin and GABA signaling in VLPO and orexin neurons contributes to iVIS in mammals remains to be elucidated.

In this work, we tested intermittent vibration in AD animals with distinct sleep disorders. Vibration reversed all sleep deficits in fly and mice models, although we noticed that sleep enhancement was much stronger in flies compared to mice. For example, the relative increase in sleep time was larger in flies, and sleep bout duration was greatly increased (enhanced sleep maintenance and potentially more deep sleep) in flies but not in mice. Moreover, we found that iVIS induced by 30 s/1min protocol was long‐lasting for days in AD flies, with reduced Aβ depositions and reversed memory deficits. Although several studies have shown several other treatments exerted potential in improving the sleep quality of old adults, including bright light therapy and physical exercise,^[^ ^29^, ^67^ ^]^ our data support gentle vibration in certain conditions could be used as an alternative approach in treating sleep disorders and related symptoms. Note that our study does not provide direct evidence that links iVIS to the observed functional improvements in AD models, although it likely contributes to the Aβ clearance and cognitive enhancements. Given various potential physiological impacts of vibration stimuli beyond iVIS (such as social activity), it's essential to examine their potential involvements and contributions in future research. Understanding the synaptic, cellular, and circuit bases by which vibration enhances sleep has important implications for sleep innervations in patients with sleep and neurological disorders. Therefore, detailed animal and clinical studies are also needed to identify whether vibration could improve sleep quality as well as alleviate disease‐related symptoms in mammals.

## 4 Experimental Section

### Animals

Flies were raised on standard cornmeal‐yeast‐sucrose medium food and kept in a 12h/12h LD cycle at room temperature until behavioral experiments. The following strains, obtained from the Bloomington Drosophila Stock Center (BDSC) or Tsinghua University (THU), were used in the study: *w^1118^* (64349), *per^KG00536^* (13104), *PDF^01^* (26654), *nina^C3^* (1659), *sNPF‐Gal4* (51991), *NPF‐Gal4* (25681), *PDF‐Gal4* (6899), *Gad1‐Gal4* (51630), *AstA‐Gal4* (84593), *23E10‐Gal4* (49032), *Tdc2‐Gal4* (9313), *UAS‐tnt* (28838), *UAS‐shi^ts^* (44222), *UAS‐Gad1^RNAi^* (28079), *UAS‐Rdl^RNAi^* (31662), *UAS‐AstA^RNAi^* (THU, 2070), *UAS‐AstAR1^RNAi^* (THU, 2591), *UAS‐mCD8‐GFP* (5137), *UAS‐APP* (6700), *UAS‐CaLexA* (66542), *MB594B‐Gal4* (68255), *MB131B‐Gal4* (68265), *MB185B‐Gal4* (68267), *MB371B‐Gal4* (68383), *MB463B‐Gal4*(68370), *MB607B‐Gal4* (68256), *MB418B‐Gal4* (68322), *MB110C‐Gal4* (68262), *MB112C‐Gal4* (68263), *MB057B‐Gal4* (68277), *MB312B‐Gal4* (68314), *MB032B‐Gal4* (68302), *MB315C‐Gal4* (68316). *UAS‐Aβ42* was obtained from Dr. Yi Zhong.^[^ ^68^ ^]^ All flies used for sleep monitoring were backcrossed with the *w^1118^* strain at least 5 times to standardize the background before experiments. Males were used in the study unless otherwise specified. We added nipecotic acid (NipA, 10 mg ml^−1^, Sigma) and EOS (10 mm, Sigma) in the fly food, and the drugs were fed during the sleep recordings.

Male inbred C57BL/6N mice, aged 8–10 weeks, were purchased from Shanghai SLAC Laboratory Animal Company (China). Transgenic mice carrying five mutations associated with early onset familial AD (5XFAD mice) were also used in this work.^[^ ^69^ ^]^ The animals were housed at a controlled temperature (22 ± 1 °C) and humidity (60–70%) under a 12/12 h LD cycle with lights on at 8:00 and off at 20:00 (illumination intensity at ≈100 lux). Food and water were always given ad libitum. The WT animals were randomly assigned to experimental and control groups. All the mice experiments in this study were performed in accordance with protocols approved by the Institutional Animal Care and Use Committee of Shanghai Jiao Tong University (SYXK(SH)2011‐0112).

### Drosophila Sleep Assays

For sleep data collection, flies aged 1–3 days (unless otherwise specified) were collected and loaded into 5 × 65 mm diameter glass tubes containing 5% sucrose and 2% agar. The baseline sleep was measured for 3 days after being loaded into the tubes. Locomotor activities were recorded by the *Drosophila* Activity Monitoring System (DAMS, Trikinetics, Waltham, MA, UAS). Experiments were done at 25 °C except for the thermogenetic experiments using *UAS‐Shi^ts^* transgene. *UAS‐Shi^ts^* flies were raised at 22 °C and monitored for 3 days at 22 °C to determine the baseline sleep, and then the temperature was raised to 30 °C for *Shi^ts^* activation. Vibration was applied on the second day at 30 °C. *Drosophila* activity data (beam breaks) were binned at 1 min intervals. Sleep is defined as a period of immobility that lasts for 5 min or more, based on previously established criteria. Sleep latency was defined as the time for a fly to enter the first sleep bout after light‐on or light‐off. Morning and evening anticipation indexes were defined as the total activity 3 h before the light/dark transition divided by the total activity 6 h before the light/dark transition. Sleep analysis was performed using an R‐based software SleepyFlyR.^[^ ^70^ ^]^

For generation of gentle vibration in flies, DAM modules were mounted on custom‐made platforms with a shelf (12.65 × 4.8 cm), and the vibrating motors (Xinling Technology Co., Ltd, Guangzhou, China) were placed in the middle of the shelf as shown in Figure 1A. Each platform/motor holds two DAMs. Motors of different vibrating rates (7, 12, and 18 Hz) exhibited movement amplitude constantly at 0.2 cm. The duration and the timing of the stimulation were controlled by the LC4 light controller (Trikinetics, Waltham, MA, UAS). An electrical device, a time relay controller (MD 1M1S1, Shanghai Sieland Electric Co., Ltd, Shanghai, China) was used, for the 15s/30s stimulation. The angular velocity (deg/s) data were also collected via a data acquisition device (BWT901CL, Wit‐motion Intelligence, Shenzhen, China).

For arousal threshold assay, flies were entrained under normal LD cycle conditions with moderate light strength (≈200 Lux). On the day of the experiment, vibration was applied at ZT 0 to promote sleep, and a strong light stimulus (≈500 Lux) was provided at ZT 6 for 1 min. Both vibration and light were controlled by the LC4 light controller. Light responsiveness was measured as the changes in the number and percentage of active flies within 4 min after the flies were exposed to strong light. The raw data was processed with a custom R script.

### Mice Polygraphic Recordings and Vigilance State Analysis

An adult mouse with indicated age and genotype in the text was anesthetized with 1% sodium pentobarbital (50 mg kg^−1^, i.p.), placed on a stereotaxic frame, with its head fixed by a stereotaxic frame adaptor. The mouse was then chronically implanted with six electrodes for polysomnographic recordings (EEG), electromyogram (EMG), and grounding (reference). Briefly, 2 skull screws (electrodes, 1 mm in diameter) were inserted into the frontal skull for EEG recordings, and 2 others were inserted into the skull of the lateral parietal region for grounding. 2 electrodes (Teflon‐coated, stainless‐steel wires) were placed bilaterally into the neck muscles for EMG recordings. All the electrodes were then fixed tightly to the skull by dental cement and connected to a micro‐connector.

After surgery, the mice had a ≈10‐day recovery period and then were housed individually and habituated to the recording environment for 3 days before polygraphic recordings. A slip‐ring‐designed recording cable was used so that the movements of the mice were not restricted. Each animal was recorded continuously for 4 days starting at 20:00, the beginning of the dark period. Vibration was applied on the third day at 20:00 for 12 h during the dark period.

Cortical EEG and EMG signals were digitized at 3 KHz, filtered at 200 Hz, and further analyzed using SleepSign software (3.2.6.1404). Polygraphic recording data were automatically scored offline as wakefulness, NREM sleep, or REM sleep in 4 s epochs using SleepSign according to standard criteria. Specifically, wakefulness was defined as EEG with relatively low amplitudes and high frequency, desynchronized waves, and EMG with phasic bursts; NREM sleep criteria defined as high amplitudes, low frequency, and synchronized waves; REM sleep criteria defined as muscle atonia and exhibiting apparent theta waves. Sleep latency was defined as the total time taken for the first NREM sleep episode (lasting at least 20 s) to appear from the time of lights off and/or vibration on (at 20:00).

Mice were subjected into two different experimental protocols as described here. For protocol 1, mice housed in their home cage with sound attenuated were put on a linear‐motion platform reciprocating horizontally (SH240, Yangzhuo Biotech Co., Shanghai, China). The platform provides movement with a periodical displacement of ±2.0 cm along one axis. Four formally vibrating modes were used in the study, including 1h/2h (1 h vibration followed by 1 h rest), 5min/10min (5 min vibration followed by 5 min rest), 1min/2min (1 min vibration followed by 1 min rest) and 12h/12h (continuous vibration for 12 h). 1 Hz vibration was applied throughout the study to induce sleep according to a previous study.^[^ ^7^ ^]^ The amplitude of the movement was 2 cm (referred to as A) and the peak acceleration was determined as α_max_ = 4π^2^f^2^A. So, the peak acceleration at 1 Hz is ≈79 cm^−1^s.^[^ ^2^ ^]^ EEG and EMG waves were continuously recorded for 4 days (96 h), and vibration started at 20:00 (lights off) on the third day. EMG and EEG data from the first two days were used as baseline control, whereas data after vibration off referred to as the recovery period. Animals were vibrated under different modes for 12 h, as indicated in Figure 5. We used a within‐subject design to control the number of animals used in the study. So, to better control the order effects, the mice were randomly subjected into two groups, each with a different sequence of vibrating conditions. Each mouse was vibrated with all the aforementioned conditions, and there are 7‐day gap between each rocking experiment. For protocol 2, flumazenil (0.1 or 1 mg kg^−1^, dissolved in sterile saline; MedChemExpress, USA) was injected intraperitoneally (i.p.) 15 min before vibration onset. For baseline data, sterile saline alone (vehicle, Veh) was also injected as control.

### Long‐Term Courtship Memory Analysis

Male and female *Drosophila* were collected after eclosion within 8 h and kept individually in vials aged up to 10–15 days. Before the training experiments, the collected females (n = 8–10) were pre‐mated with matured males (n = 10, from stock) in a food vial for over 18 h till the test.

Repeat training courtship suppression assay was used to evaluate long‐term courtship memory. For training, a freshly mated female was placed with a male for 7 h from ZT 0. While another male kept alone was used as naïve control. After training, females were removed from the vials, and the males were quickly transferred (within 15 min) to the DAM2 tubes for sleep recordings during the following 24 h. Sleep recording starts at ZT 7.5. For the iVIS test, half of the males were mechanically vibrated till the test, while the others kept stationary. Then the experimental and naïve males were moved to a test chamber containing a freshly mated female with the same age as the males for 10 min videotaped testing period. Normally, a male previously rejected by a mated female would show reduced courtship bouts to another mated female due to enhanced sensitivity to volatilized cis‐vaccenyl acetate (cVA) from the female cuticle. The time spent on performing stereotypic courtship behaviors was scored and a courtship index (CI) was defined as the percentage of the total 10 min that was spent in courtship behaviors. Memory index (MI) was further defined by using means CIs from each group (with 3 naïve and 3 trained flies): MI = (CI_naïve_−CI_trained_ / CI_naïve_). A mean CI for each group was first determined, and then a MI was subsequently calculated.

### Immunohistochemistry

Brains of indicated genotypes were dissected in phosphate‐buffered saline (PBS) buffer (1×, pH 7.4) and fixed in 4% paraformaldehyde for 20 min and then were washed by PBS for 10 min, 3 times. Brains with 5% goat serum for 2 h were blocked and stained with 6E10 anti‐Aβ42 (1:200, SIG‐39320, BioLegend, CA, USA) in PBST buffer (0.1% Tween‐20 in 1× PBS) on a shaker at 4 °C for incubation overnight. Brains were washed (10 min, 3 times) by PBST and followed by secondary antibody (rhodamine‐conjugated goat anti‐mouse, 1:500) incubation for 2 h at room temperature. Fluorescence images were taken by a Leica SP8 confocal microscope. DAPI was included for Aβ42 staining during brain mounting. Aβ fluorescence in mushroom body areas were analyzed, as reported previously.^[^ ^52^ ^]^ Gray values of Aβ fluorescence were measured by subtracting the background values using ImageJ software.

### Patch‐Clamp Recordings

Transgenic males, including *23E10‐Gal4>UAS‐mCD8‐GFP* and *Tdc2‐Gal4>UAS‐mCD8‐GFP*, aged 6–7 days were used for patch‐clamp recordings from GFP‐labeled dFB neurons and GFP‐labeled anterior superior medial (ASM) neurons in the medial protocerebrum, respectively. Flies were anesthetized by putting them on ice for 2 min. Then, the brains were isolated from heads in *Drosophila* culture medium (GIBICO) and moved to a recording chamber with external solution (pH ≈7.2) containing 115 mm NaCl, 2 mm KCl, 6 mm MgCl_2_, 1 mm CaCl_2_, 5 mm glucose, 20 mm NaHCO_3,_ and 2 mm NaH_2_PO_4_. The brains were fixed by a homemade nylon grid glued to a platinum frame. External solution bubbled with carbogen (5% oxygen and 95% carbon dioxide) was constantly perfused into the recording chamber at a rate of 1 mL min^−1^ throughout the recordings. GFP‐labeled cells were visualized under a 40× objective. The glial sheath above the targeted GFP‐labeled cells was gently removed with sharp glass electrodes before recordings. Tetrodotoxin (TTX) (1 mm) and Tubocurarine chloride (30 mm) were added to the external solution to record GABA receptor‐mediated currents (with the chloride equilibrium potential ≈0 mV) in ASM cells at voltage‐clamp mode (holding at ‐90 mV), as reported previously.^[^ ^46^, ^71^ ^]^

Electrodes were filled with an internal solution containing: K‐gluconate,125 mm; KCl, 20 mm; Mg‐ATP, 4 mm; Na‐GTP, 0.5 mm; HEPEs, 10 mm; EGTA, 1.1 mm; MgCl_2_, 2 mm; CaCl_2_, 0.1 mm, with pH at 7.2. 145 mm KCl was used in initial recordings to better visualize the Cl^−^ currents. 600 mg mL^−1^ Amphotericin‐B (Sigma–Aldrich) was also added in the internal solution for perforated‐patch. No current (0 pA) was injected for current‐clamp recordings in dFB neurons. To test the potential effects of iVIS on neuronal activities, flies were vibrated from ZT 0 to ZT 4 for 4 h and then evaluated the excitability of dFB neurons by injection of a series of current steps (from −10 to 50 pA, 10 pA increments per step). Data were collected between ZT 4 and ZT 5, and data was discarded if the resting potentials were not stable throughout the recordings. ASM neurons were recorded during the light period (ZT –ZT12). Recordings were acquired using an Axopatch 200B amplifier, and sampled with Axon Digidata 1550. Signals were digitized at 5 kHz and filtered at 2 kHz, and data were further analyzed using pClamp 10 software (Molecular Devices Corp.). Input resistance was calculated as the maximal voltage change divided by a small hyperpolarizing current injection by a step of 150 ms duration.

### RNA‐Sequencing

For the RNA‐sequencing assay, two groups of *w^1118^* flies (baseline and 3‐h vibration) were collected at ZT 3 and sequenced together aged 5–7 days to avoid possible batch effects. Duplicate samples were assayed for each group, and 100 adult male heads were isolated for each sample. Total RNA was extracted by UNlQ‐10 Column Trizol Total RNA Isolation Kit (Sangon Biotech) and DNA was digested using DNaseI (BBI). Briefly, Oligo dT magnetic beads were used to purify and enrich RNA samples and stored the samples at −80 °C until the commencement of RNA‐sequencing.

cDNA libraries were prepared using the Illumina TruSeq stranded mRNA library prep kit. Sequencing was performed on the prepared library with the Illumina platform and paired‐end (PE) strategy. Clean reads were obtained from raw reads from the Illumina platform by removing contaminating sequences, read duplicates, low‐quality reads, etc. Based on clean reads, the transcriptome sequencing information analysis process mainly contains three steps: i) data quality control; ii) data comparison analysis, and iii) transcriptome in‐depth analysis such as Multi‐platform Gene Ontology (GO) annotation. Statistical analysis was performed, and differentially expressed genes (DEG) with statistical significance were selected through volcano plot filtering with the criteria including |log_2_‐fold change| ≥ 0.58 (≥ 1.5‐fold changes) and false discovery rate (FDR) < 0.05.

### Quantitative (q) Real‐Time PCR

For qPCR assay, two groups of *w^1118^* flies (baseline and 3‐h vibration) were collected at ZT 3 and were frozen in dry ice. Total RNA was isolated from 50 fly heads for each sample with Trizol Reagent, and then all the samples were collected and processed independently. cDNA was synthesized from the total RNA using oligo (dT)_20_ primer and Maxima Reverse Transcriptase (Thermo Scientific). The primers were 5′‐CGCCTGCCGGTCTATAACTT‐3′ and 5′‐CTTGTTCTGTCGGCCAGGTC‐3′ for *AstA*, 5′‐CATCGCCGGAATCTACACCAC‐3′ and 5′‐GCGTGAATCTTGTCCATTTGCT‐3′ for *bgm*, 5′‐ ACTCGCGCAGCAGATAAAGTC ‐3′ and 5′‐ CCCAATTTGGATAGGTGCTCG‐3′ for *Acer*; 5′‐CCGCTTCAAGGGACAGTATCT‐3′ and 5′‐CGATCTCGCCGCAGTAAA‐3′ for housekeeping gene *rp49* for reference. Relative expression of each gene was normalized against the rp49 by using the 2^−ΔΔCT^ calculation method,^[^ ^72^ ^]^ ΔΔCT = CT_(target, test)_−CT_(rp49, test)_−[CT_(target, calibrator)_−CT_(ref, calibrator)_] to evaluate the efficiency of each reverse transcription.

### Statistical Analysis

The electrophysiological recording data were analyzed utilizing pClamp 10 software (Molecular Devices Corp.) and SleepSign software (3.2.6.1404). Staining images were analyzed using ImageJ (FIJI, NIH). Experiments and data analyses were conducted in a blinded manner wherever feasible. The number of animals and cells recorded is provided in figure legends. Data were expressed as mean ± SEM. Normality was tested using the Kolmogorov–Smirnov normality test. Statistical analysis was mostly performed by GraphPad Prisms 9. Unpaired student's t‐test was performed by comparing between two groups. One‐way ANOVA followed by post hoc Tukey tests were used to test hypotheses involving multiple groups. For comparisons of non‐normally distributed data (sleep bout number and duration), Mann‐Whitney U tests were performed using SPSS software 26.0 (IBM). Statistical significance was set at p < 0.05

## Conflict of Interest

The authors declare no conflict of interest.

## Author Contributions

Y.M. and Y.Z. contributed equally to this work. Y.P. supervised the project and conceived the study. X.X., Y.M., Y.Z., G.H., Z.X., and Y.P. designed the experiments. Y.M., Y.Z., and Y.P. executed the experiments and conducted statistical analysis. X.X. G.H. and Y.P. provide critical resources. Y.P. wrote the paper with contributions from all authors.

## Supporting information



Supporting Information

Supplemental Table 1

Supplemental Table 2

## Data Availability

The data that support the findings of this study are available from the corresponding author upon reasonable request.
