# Supplementary material for: Intermittent Vibration Induces Sleep via an Allatostatin A‐GABA Signaling Pathway and Provides Broad Benefits in Alzheimer's Disease Models
Source: Adv Sci (Weinh). 2024 Dec 10;12(5):2411768. doi: 10.1002/advs.202411768 (PMC11791986; doi:10.1002/advs.202411768)
Supplement: Supplementary file 1 — Supporting Information [file ADVS-12-2411768-s003.docx]

**
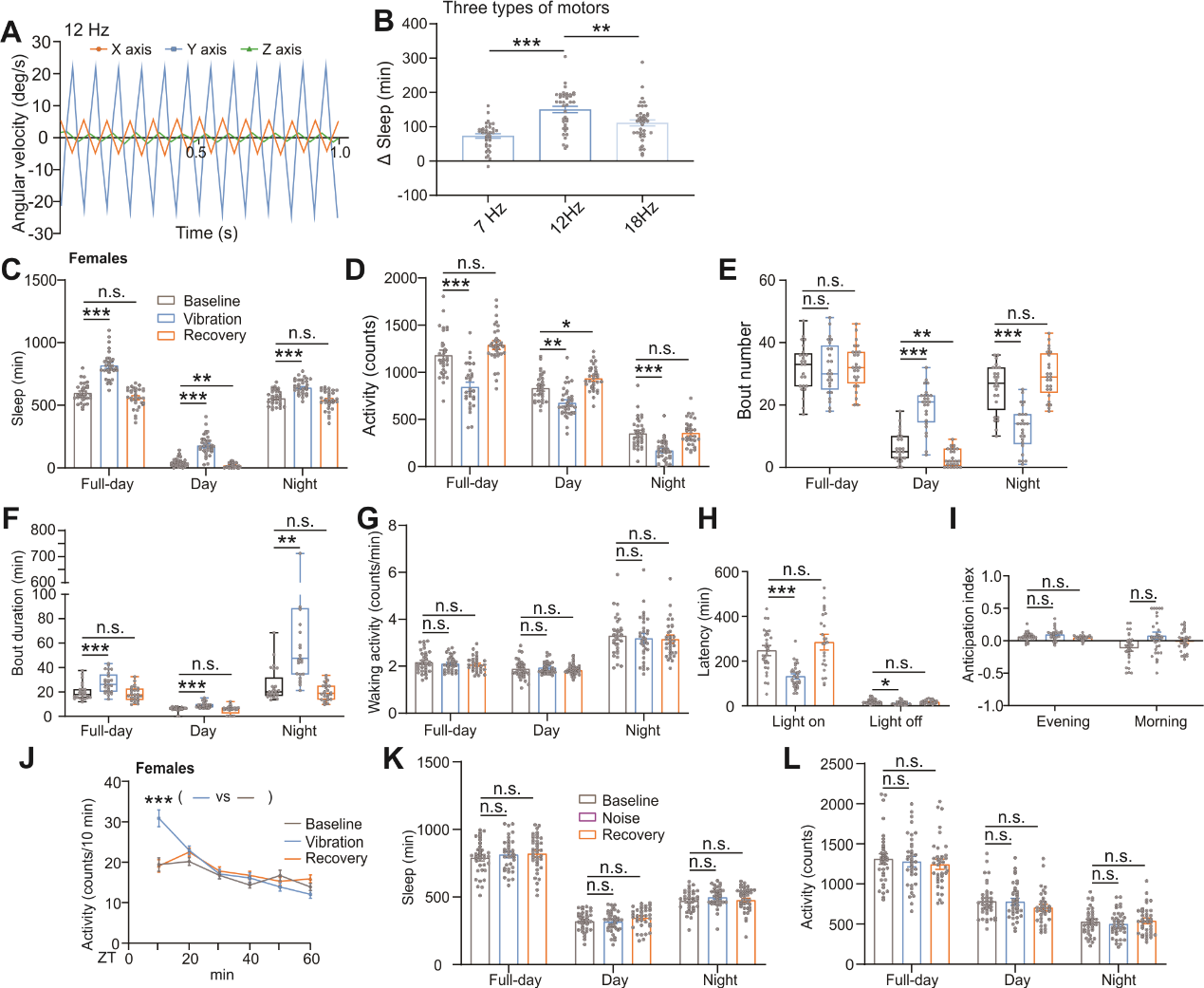
Figure S1. iVIS in *Drosophila*.**

(A) Measurement of angular velocity (deg/s) produced by a vibrating motor at 12 Hz. The major velocity was on Y axis, almost no movement on X and Z axes.

(B) Sleep change during vibration in different conditions, including 7 Hz, 12 Hz and 18 Hz. n = 35- 41 for each group. Vibration was on for 1 day.

(C-G) Quantification of full-day, day and night sleep time, activity counts, bout number, bout duration and waking activity in females as shown in Figure 1F, G. There is no difference in waking activity during baseline, vibration and recovery periods.

(H and I) Quantification of sleep latency and anticipation index in females as shown in Figure 1F, G. Sleep latency and anticipation index for both light on and off were shown.

(J) Activity counts for 60 min after vibration on in females.

(K and L) Quantification of full-day, day and night sleep time and activity counts of males in baseline, noise and recovery periods.

**
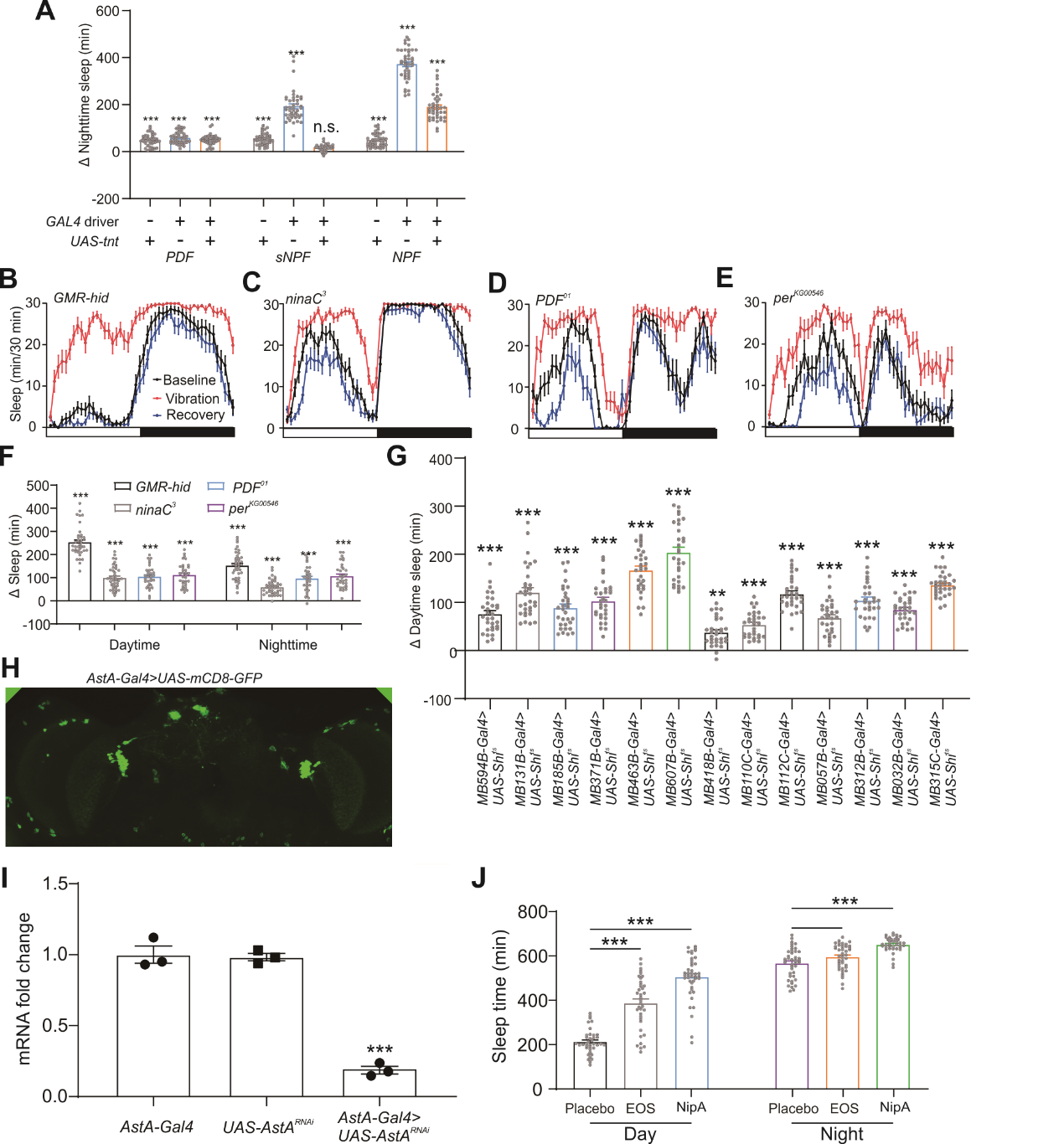
Figure S2. Transgenic lines screened for iVIS.**

(A) Quantification of daytime sleep changes as shown in Figure 2A and 2B. Blocking activities of PDF, sNPF or NPF neurons show no obvious effects on iVIS.

(B-E) Conventional sleep profiles of the mutants of indicated genotypes during baseline, vibration and recovery periods in 12h LD conditions. n = 30-46 flies for each line.

(F) Quantification of sleep changes during daytime and nighttime in lines as shown in (B-E).

(G) Quantification of daytime sleep changes of indicated lines during vibration. Note that vibration induced daytime sleep in all the lines tested. n = 34-42 flies for each line.

(H) Neurons labeled by GFP in *AstA-Gal4>UAS-CD80GFP* line. Scale bar, 100 μm.

(I) Shown are RT-qPCR analysis for mRNA levels of *AstA* in AstA-Gal4>UAS-AstA^RNAi^ and control lines. n = 3 independent qPCR for each case.

(J) Quantification of sleep time in placebo and drug treatments (EOS or NipA) conditions, related to Figure 2L.

**F
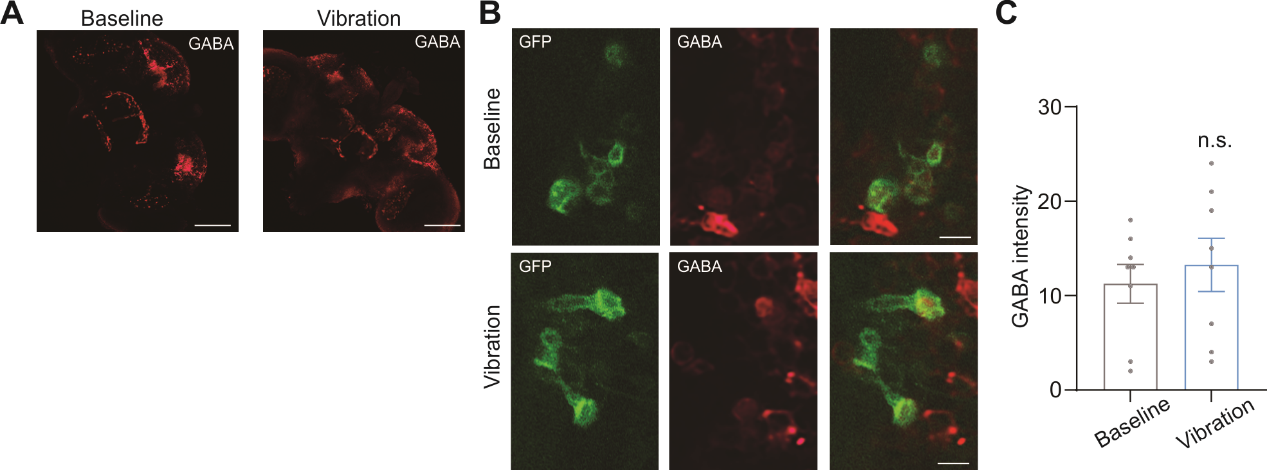
igure S3. GABA staining in entire brains and dFB neurons.**

(A) Representative GABA staining whole brain images, with a focus on the posterior brain. We noticed that GABA was stained across the brain in baseline condition. Vibration may altered GABA levels in some specific brain regions (such as ASM as discovered in the paper), but increase in GABA levels may not be a widespread phenomenon. Scale bars, 100 μm.

(B) Brains of the *23E10-Gal4>UAS-CD8-GFP* flies dissected at ZT3 including baseline (Base, H) and vibration (Vib, I) for 3 h from ZT 0~3 are stained with GABA (red). Scale bars, 10 μm.

(C) Bar graphs represent relative GABA intensity as shown in (B). n = 8 brains for each case.

**
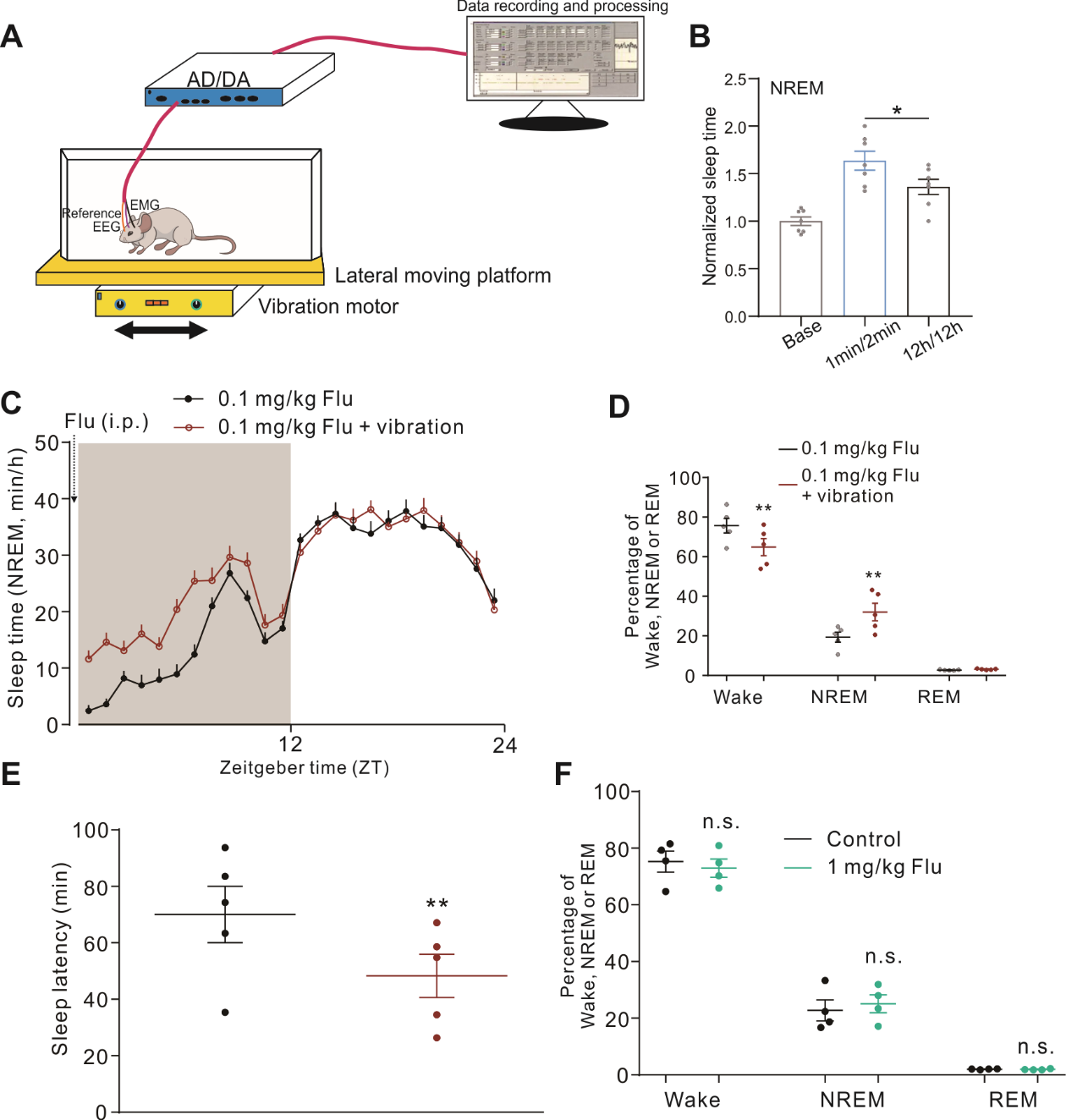
Figure S4. iVIS partially blocked by Flu in mice.**

(A) Schematic representing experimental protocol used to measure the sleep of individually housed male mice. The mice were placed on a laterally moving platform. Either continuous or intermittent vibration were applied.

(B) Time course for NREM sleep in baseline or vibration (during dark period at 1 Hz) in the groups of vehicle (Veh) or Flu (0.1 mg/kg) as indicated in the figure.

(C) Percentage of time in NREM sleep, REM sleep, or wake state during dark period in baseline or vibration conditions as shown in B.

(D) Quantification of sleep latency in baseline or vibration conditions as shown in B.

(E) Percentage of time in NREM sleep, REM sleep, or wake state during dark period in control (Veh) or 1 mg/kg Flu injection.

**
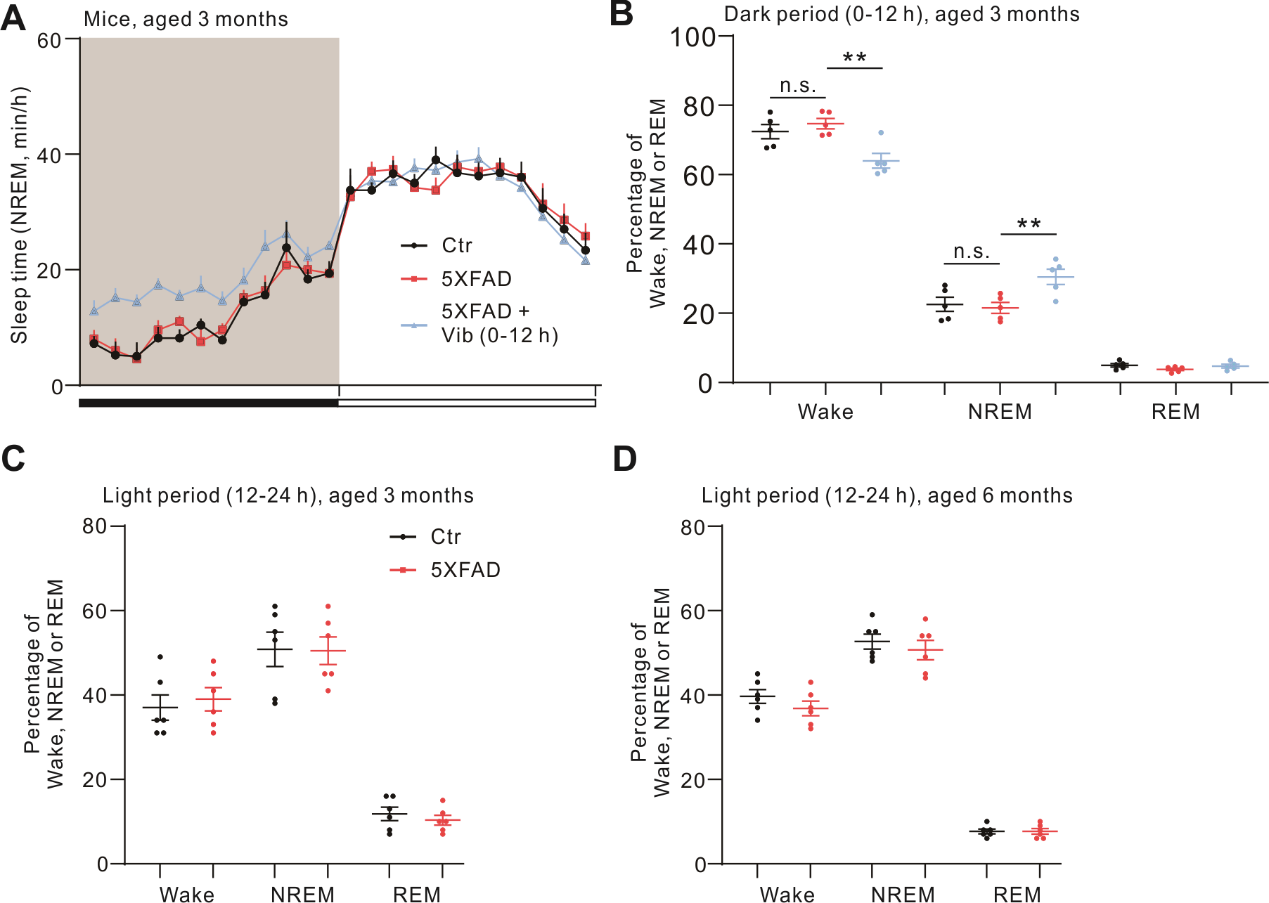
Figure S5. iVIS in 5XFAD mice.**

(A) Time course for NREM sleep (Mean, min/hour) during baseline or vibration (Vib) for control (Ctr) and 5XFAD transgenic mice as indicated. Intermittent vibration was applied for 12 hours (h) during dark phase.

(B) Percentage of time in NREM sleep, REM sleep, or wake state during dark period in lines as shown in (A).

(C, D) Percentage of time in NREM sleep, REM sleep, or wake state during light period in Ctr and 5XFAD mice aged 3 months (C) or 6 months (D).

**
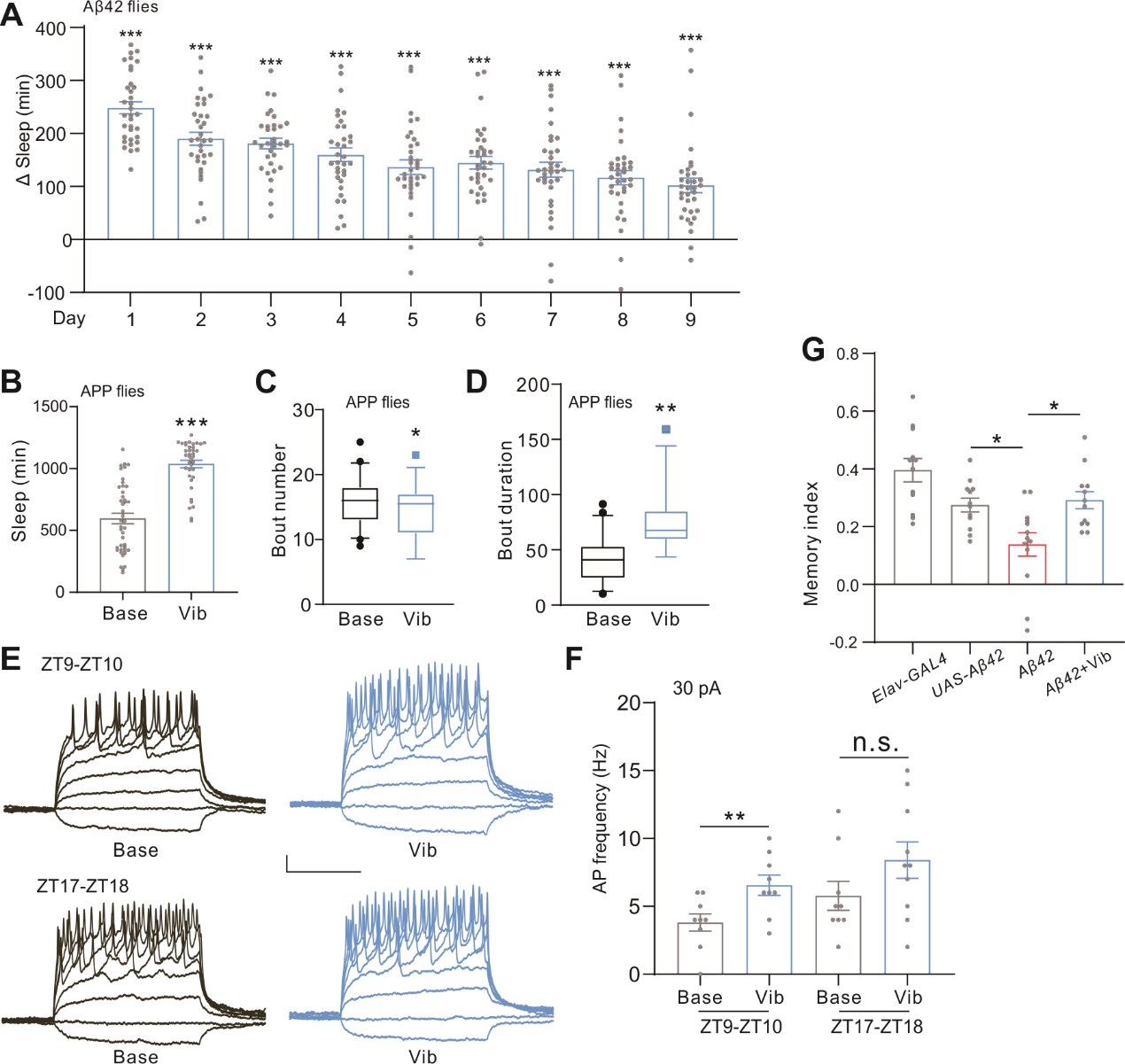
Figure S6. Vibration reverses memory defects in AD flies.**

(A) Quantification show that intermittent vibration (30s/1min) causes long-lasting (up to 9 days) sleep enhancement in Aβ42 flies (*Elav-Gal4>UAS-Aβ42*). n = 34 flies.

(B-D) Quantification of sleep time (B), bout number (C) and duration (D) in baseline (Base) and vibration (Vib) conditions following courtship training as shown in Figure 7D of APP flies (*Elav-Gal4>UAS-APP*). n = 38-43 flies for each case.

(E) Representative voltage responses to 500 ms current steps (from -10 to 60 pA, 10 pA increment per step) of *23E10-Gal4>UAS-mCD8-GFP* at baseline and vibration conditions. Recordings were conducted during ZT9-10 and ZT17-18.

(F) Quantification of firing rates as recorded in (E). n = 8-10 cells for each case.

(G) Memory index (MI) of the lines as indicated. n = 12-13 groups for each case.
